# A New Disease of the Gums

**Published:** 1860-10

**Authors:** 


*A New Disease of the Gums.*—M. Marchal has lately given an account, before the Academy of Sciences, Paris, of an undescribed disease of the gums. It first loosens the teeth, and, subsequently, causes them to fall out. Hence the disease has been characterized as *expulsive inflammation of the gums.* It is generally suppurative, and sometimes ulcerous. The remedy is a local application of iodine.

We shall furnish a translation of M. Marchal's memoir in the January number.

